# New Powers for Urban and District Councils

**Published:** 1918-01-26

**Authors:** 


### SOME PROBLEMS OF TO-DAY.

### New Powers for Urban and District Councils.

By A SOCIAL WORKER.

One result of the delay in the Education Bill seems to have been to stimulate other effort on behalf especially of the tiny children, ex-babies and toddlers who, it was hoped, would be gathered in at once to the shelter of the nursery school. The deputation from the Associations of Urban and District Councils, representing some 800 of the former and 600 of the latter, which waited on Mr. Hayes Fisher, received a sympathetic and to some extent a hopeful reply. It is true that nothing could more clearly show how much we need a Ministry of Health than the revelation of difficulties under which the Local Government Board has to act, if it does act, the collisions between authorities, and the overlapping. The deputation earnestly assured Mr. Fisher that the Councils were most anxious to use the powers asked for, which are, after all, only those already possessed by Ireland and Scotland, to meet the pressing needs of mothers and infants. A Bill for this very extension, brought in by Mr. Walter Long in 1915, was wrecked by these rivalries. Yet Lord Rhondda has lately said that he believes a thousand lives a week might be saved by such measures, and, seeing what has been done in places where a high infant-mortality rate, with its invariable high rate of damaged lives, has been vigorously attacked, we can readily believe this. The representative of Swinton and Pendlebury Council emphasised the need for vigour in his relation of his Council's efforts to reduce the outrageous rate of 200 per thousand. Hornsey, let us remember, though a district of London, has lowered its rate to a little over 70 in 1,000 by diligent effort. A patent scandal in the Swinton area was the permission granted to an ice-cream seller to use 1 cwt. of sugar monthly, his *weekly* consumption of milk amounting to no less than 96 quarts.

### Milk Shortage and Sweet-making.

It is sometimes urged in defence of our still-open sweet-shops that much of the underfeeding of children is remedied by the sugar they get in this way. But we can hardly regard it as satisfactory that milk shortage should be officially esteemed as compensated for by hokey-pokey sucked down in the street. The same speaker pleaded that what is needed is really to place the working-man's son and daughter in as good a position as the Poor-Law child. There are certainly districts in England —there is one a few miles from London—where bitter jealousy has been stirred by the conditions observable in the Poor-Law village, including the pocket-money available for sweets.

Mr. Hayes Fisher has stated that he feels the municipal control of milk, both as to purity and as to the preference for mothers and babies in its distribution, to be a matter for immediate legislation. His promise to obtain from the War Cabinet leave to introduce a Bill conferring the desired powers, which would of course include authority to deal with the two great questions of midwifery and housing, is something gained.

### Housing the Large Family.

Do most of us yet realise how inseparable these two questions really are? Encouragement, Mr. Hayes Fisher confessed, needs to be given to the increase of population. He might have said *the removal of discouragement*: "'Tisn't everyone wants me and my eight." "No one don't seem anxious for us and our five," two different mothers have told the present writer in the last few weeks. It is no wonder if the working man's wife is perplexed when the literary columns of her newspaper refer to the children as "their country's most precious asset," and the advertisement page calls them "encumbrances." It is the housing of the *large* family, with proper provision for health and cleanliness, chiefly a sufficient supply of clean air and clean water, that is in question. Meantime the mother, exhorted unceasingly to bring up her children better, has now grasped the idea that she could do more for a few, and that it is not necessary to have so many. A woman said the other day to a trusted friend, with a glance at her waiting cradle, "I haven't tried nothink yet meself, but my sister's that comfortable with on'y three. . ." It is not entirely easy to cut the Gordian knot of her difficulty with the knife of religion or morality, and say that parental self-control is the only permissible limitation. People in easy circumstances can hardly realise that sevenfold heating of the furnace to a neglected wife, the sight of her children hungry and cold because their father's earnings have been diverted elsewhere.

### The Mother's Best Place.

To an improved midwifery service and the provision of skilled help in the home there must be the warmest welcome, but as regards the dictum that working-class homes are not "ideal" for the purpose of confinement, we can only hope that it will not be pressed too far. Most of us have to stop short of the ideal in most things, and while for abnormal cases hospital accommodation is very desirable for several reasons, in any case where the mother can make simple but sufficient provision, and proper help is available to give her the days of needed rest, she is far happier in her own surroundings. Those who can plead that "a single woman is better off in the workhouse infirmary" speak without experience. Even near relations cannot always be left in charge. The writer remembers the bitter vexation of a respectable but very poor Irishwoman whose own mother cut the children's best blanket into a petticoat for herself while "taking care" of the home.

### The Lady Visitor.

Social problems, in fact, cannot be settled by a stroke of the pen. "Increase the maternity benefit," say some, "and all is done." But there are homes in which debt is incurred on the strength of the present thirty-shilling benefit. A not at all impossible result in such families might be "Mother's got a new baby, *so* we've got a gramophone." The sneer which in some quarters is being aimed at the lady visitor of poor homes is a hollow reproach. It is by the spade work of such visiting that the ground has been prepared for the sowing of a new crop of wise measures. And much patient and persevering detail work will be needed before the country can reap the full gain. "Blessed," it was long ago said, "is he that *considereth* the poor."

